# Acceleration of Brain Atrophy and Progression From Normal Cognition to Mild Cognitive Impairment

**DOI:** 10.1001/jamanetworkopen.2024.41505

**Published:** 2024-10-30

**Authors:** Yuto Uchida, Kei Nishimaki, Anja Soldan, Abhay Moghekar, Marilyn Albert, Kenichi Oishi

**Affiliations:** 1 Department of Radiology and Radiological Science, Johns Hopkins University School of Medicine, Baltimore, Maryland; 2 Department of Neurology, Johns Hopkins University School of Medicine, Baltimore, Maryland; 3 The Richman Family Precision Medicine Center of Excellence in Alzheimer’s Disease, Baltimore, Maryland

## Abstract

**Importance:**

It remains unclear which risk factors accelerate brain atrophy along with a progression from normal cognition to mild cognitive impairment (MCI).

**Objective:**

To examine risk factors associated with the acceleration of brain atrophy and progression from normal cognition to MCI based on long-term longitudinal data for middle-aged and older adults.

**Design, Setting, and Participants:**

Data for this cohort study were extracted from the Biomarkers for Older Controls at Risk for Dementia (BIOCARD) cohort, initiated at the National Institutes of Health from January 1, 1995, to December 31, 2005, and continued at Johns Hopkins University from January 1, 2015, to October 31, 2023. All participants were cognitively normal at baseline. The participants whose structural magnetic brain imaging (MRI) of the brain and cerebrospinal fluid (CSF) measures were available for over 10 years were included.

**Exposures:**

Longitudinal structural MRI of the brain and measurement of CSF biomarkers for Alzheimer disease pathology (ratio of amyloid β peptide 42 [Aβ_42_] to Aβ_40_, tau phosphorylated at threonine 181, and total tau).

**Main Outcomes and Measures:**

Annual change rates of segmental brain volumes, Kaplan-Meier survival curves plotting time to event for progression to MCI symptom onset, and hazard ratios (HRs) determined by Cox proportional hazards regression models.

**Results:**

A total of 185 participants (mean [SD] age, 55.4 [8.4] years; 116 women [63%]) were included and followed up for a maximum of 27 years (median, 20 [IQR, 18-22] years). The groups with high levels of atrophy in the white matter and enlargement in the ventricles had an earlier progression from normal cognition to MCI symptom onset (HR for white matter, 1.86 [95% CI, 1.24-2.49]; *P* = .001; HR for ventricles, 1.71 [95% CI, 1.19-2.24]; *P* = .009). Diabetes was associated with progression to MCI (HR, 1.41 [95% CI, 1.06-1.76]; *P* = .04), as was a low CSF Aβ_42_:Aβ_40_ ratio (HR, 1.48 [95% CI, 1.09-1.88]; *P* = .04), and their combination had a higher HR of 1.55 (95% CI, 1.13-1.98]; *P* = .03), indicating a synergic association of diabetes and amyloid pathology with MCI progression.

**Conclusions and Relevance:**

In this cohort study of middle-aged and older adults, higher rates of volume change in the white matter and ventricles, along with the presence of diabetes and a low CSF Aβ_42_:Aβ_40_ ratio, were identified as important risk factors for the progression to MCI. These results support the importance of identifying individuals who have accelerated brain atrophy to optimize preventive strategies for progression to MCI.

## Key Points

**Question:**

What factors are associated with the acceleration of brain atrophy and progression to mild cognitive impairment (MCI) based on long-term longitudinal data for individuals with normal cognition at baseline?

**Findings:**

In the cohort study, 185 participants with normal cognition underwent a mean follow-up of 20 years with brain magnetic resonance imaging scans. Type 2 diabetes and abnormal amyloid-β concentration in the cerebrospinal fluid were associated with accelerated brain atrophy and an earlier progression to MCI.

**Meaning:**

These results support the importance of identifying individuals who have accelerated brain atrophy to optimize strategies to prevent MCI.

## Teaser

This cohort study examines risk factors associated with the acceleration of brain volume change and progression from normal cognition to mild cognitive impairment among middle-aged and older adults.

## Introduction

Numerous structural magnetic resonance imaging (MRI) studies across the human lifespan have reported cross-sectional data,^1^ whereas longitudinal MRI data are largely limited to less than a decade.^2,3,4,5,6,7^ Although the execution of the longitudinal study design requires substantial costs and efforts, it has the potential to offer distinct advantages over the cross-sectional study design. For instance, cross-sectional structural MRI studies are unable to quantify the variability in brain atrophy rates among individuals in categorized groups, since these studies operate under the assumption that pathophysiological effects on brain morphology remain consistent across individuals.^8^ Meanwhile, longitudinal MRI studies enable us to track changes in brain morphology over time on an individual basis, which facilitates the differentiation between interindividual and intraindividual variations and leads to more accurate estimations of rates of brain atrophy.^2,9,10^

Due to the paucity of long-term observational studies of structural MRI of the human brain over 10 years, it is unclear whether the annual rates of change of brain volumes are affected by vascular risk factors and are useful in estimating the progression from normal cognition to mild cognitive impairment (MCI) in aging individuals. Several previous longitudinal studies, which followed 2 time points of longitudinal study design or less than 10 years,^11,12,13,14,15,16,17^ found associations of brain characteristics with vascular risk factors. Specifically, hypertension, dyslipidemia, and smoking were associated with increases in white matter hyperintensity volumes and asymptomatic infarctions, whereas type 2 diabetes was associated with brain atrophy and subsequent cognitive decline. For example, the Atherosclerosis Risk in Communities Neurocognitive Study^18^ found that individuals with diabetes and without dementia in midlife had as much as a 2-fold higher risk for brain atrophy and cognitive impairment, adjusted for age, sex, race and ethnicity, and educational level.^12,19^ However, some longitudinal cohort studies^20,21^ showed no direct associations of diabetes with the rate of brain atrophy. The limited follow-up period and number of MRI scans in these studies may have contributed to these discrepancies, since diabetes is associated with greater cognitive decline over 10 years or more.^22^ Moreover, it is now widely recognized that amyloid-β (Aβ) plaques, one of the hallmarks of Alzheimer disease, begin to accumulate in the brain when individuals have normal cognition, as early as midlife.^23^ Therefore, it is essential to include Aβ biomarkers as one of the baseline measures to understand the associations between diabetes and Aβ pathology.^24^

We hypothesized that annual rates of change of segmental brain volumes would be associated with vascular risk factors among middle-aged and older adults and that these trends would be associated with the progression from normal cognition to MCI. The Biomarkers for Older Controls at Risk for Dementia (BIOCARD) cohort^25^ is well-suited to test this hypothesis, as it includes individuals who were longitudinally followed up with detailed clinical and cognitive assessments, brain MRI scans, and cerebrospinal fluid (CSF) measures of Alzheimer disease biomarkers for a mean (SD) of 20.2 (3.0) years.^26^ Using the BIOCARD cohort, this study examined risk factors for the acceleration of brain atrophy and progression from normal cognition to MCI.

## Methods

### Participant Selection and Consensus Diagnosis

The present study followed the Strengthening the Reporting of Observational Studies in Epidemiology (STROBE) reporting guideline and included data from participants in the BIOCARD cohort, all of whom had normal cognition at baseline. Among them, we selected the individuals for whom structural MRI of the brain and CSF measures were available for over 10 years from enrollment at the National Institutes of Health (NIH) to subsequent follow-up at Johns Hopkins University (JHU). This study was approved by the Johns Hopkins Institutional Review Board, and all participants who agreed to continued follow-up signed consent forms approved by the board. Details of the study design and recruitment of participants in the BIOCARD cohort are described in the eMethods in Supplement 1.

### Vascular Risk Assessments

Vascular risk factors, including hypertension, dyslipidemia, diabetes, and smoking, were obtained through self-reports collected during a medical history interview from each participant through all visits or medical records. Details of the definition of vascular risk at baseline evaluation, procedures for changes during the follow-up period, and consideration of treatments for vascular risk factors throughout the follow-up period are described in the eMethods in Supplement 1.

### MRI Analyses

The MRI scans acquired at the NIH from January 1, 1995, to December 31, 2005, included the spoiled gradient echo sequence with both sagittal and coronal section orientations using a 1.5T scanner (GE HealthCare), whereas those acquired at JHU from January 1, 2015, to October 31, 2023, included the magnetization-prepared rapid acquisition gradient echo sequence with a sagittal section orientation using a 3T scanner (Koninklijke Philips NV). Details of the scan parameters, brain parcellation using a deep learning–based method (OpenMAP-T1),^27^ and volume measurements are described in the eMethods in Supplement 1.

### Annual Rates of Change in Brain Volumes

As the longitudinal changes in the brain volume measurements within individuals were imaged under different software upgrades and scanner replacements during the study period, potential differences derived from imaging protocols were calibrated using a method for harmonizing longitudinal data (longitudinal ComBat).^28^ Then, a linear-fitting regression model was run for each participant to estimate the rate of change in the brain volume measures over time, using the brain volume measures as a dependent variable and the age of the participant at each MRI scan as an independent variable. The slope of the regression line was used as the estimate of annual rates of change in brain volumes.

### CSF Assessments

The concentrations for the ratio of amyloid β peptide 42 (Aβ_40_) to Aβ_42_, tau phosphorylated at threonine 181 (p-tau181), and total tau (t-tau) were measured. Details of CSF assessments are described in the eMethods in Supplement 1.

### *APOE* Genotype

An *APOE* ε4 carrier status was assigned as follows: individuals carrying at least 1 ε4 allele were coded as 1, while noncarriers were coded as 0. Details of how to determine the *APOE* genotypes are described in the eMethods in Supplement 1.

### Statistical Analysis

Eligible participants’ clinical characteristics were descriptively extracted. Continuous variables were expressed as mean (SD), median (IQR), or both based on the normality of data distribution. Categorical variables were expressed as numbers (percentages). These values were compared between the participants with continued normal cognition and those with progression to MCI using an independent *t* test, Wilcoxon rank-sum test, or χ^2^ test as appropriate.

For visualization purposes, longitudinal changes in segmental brain volumes corrected for intracranial volumes were plotted for individuals divided into the groups with normal cognition and progression to MCI against age and years since the first MRI scan. Differences in annual rates of change in the brain volumes as functions of vascular risk factors (hypertension, dyslipidemia, diabetes, and smoking), CSF biomarkers (Aβ_42_:Aβ_40_ ratio, p-tau181, and t-tau), and *APOE* ε4 status were evaluated using an analysis of covariance adjusted for age, sex, and educational level.

Gaussian mixture models^29^ were applied to the annual rates of change in the brain volumes for the differentiation of interindividual variations. Participants were divided into groups with high and low levels of atrophy according to the rates of change in volumes of the cortical gray and white matter. Similarly, participants were divided into groups with high and low levels of enlargement based on mixture modeling of the ventricle and sulcus volumes. Additionally, change-point analyses^30^ were conducted to identify the age when rates of change in brain volumes were accelerated.

Kaplan-Meier survival curves were separately plotted for the groups with high and low levels of atrophy of the cortical gray matter and white matter and for the groups with high and low levels of enlargement of the ventricles and sulci. Log-rank tests were performed between the groups with high and low levels of atrophy or enlargement of each brain structure. Then, a multivariable Cox proportional hazards regression model for estimating the progression to MCI symptom onset was used to determine hazard ratios (HRs) for independent variables, including demographic characteristics, high annual rates of change in the regional brain volumes, presence of vascular risk factors, positivity of CSF measures, and *APOE* ε4 status. A 2-way interaction between diabetes and low Aβ_42_:Aβ_40_ ratio was also included in this model based on our a priori hypothesis of a synergic association of diabetes and amyloid pathology with MCI progression. The time to event (progression to MCI onset) was measured from the age of the first MRI scan to the age of the latest visit supporting the onset of MCI symptoms. Censoring time was measured from the age of the first MRI scan to the age of the last completed follow-up visit. Before analyzing, the continuous variables were converted into *z* scores, calculated from the mean and SD of the participants at the first visit included in the present study.

All statistical analyses were performed using Python, version 3.9 (Python Software Foundation), with a 2-sided α = .05 indicating statistical significance. Reported results include 95% CIs when applicable.

## Results

### Study Participants

The participants’ characteristics are shown in Table 1. Overall, 185 participants, aged 20 to 76 years at baseline (mean [SD] age, 55.4 [8.4] years; median age, 55 [IQR, 52-60] years; 116 women [63%] and 69 men [37%]), were included and followed up for a maximum of 27 years (mean [SD], 20.2 [3.0] years; median, 20 [IQR, 18-22] years). Among them, 60 participants experienced progression to MCI during the follow-up period, including 8 who developed dementia after their MCI symptom onset. The total number of MRI scans was 951 and the median number per participant was 5 (IQR, 4-6). The number of MRI scans obtained at both the NIH and JHU each year is listed in the eTable in Supplement 1. Significant differences were found between the participants with continued normal cognition and those with progression to MCI in age at baseline MRI scans (mean [SD], 53.1 [7.8] vs 57.2 [8.9] years), diabetes (8 of 125 [6%] vs 11 of 60 [18%]), and positivity of a low CSF Aβ_42_:Aβ_40_ ratio (7 of 125 [6%] vs 10 of 60 [17%]).

**Table 1.** Clinical Characteristics of Study Participants

Characteristic	Participant group		
	All eligible	Continued normal cognition	Progression to MCI
All, No. (%)	185 (100)	125 (68)	60 (32)
MRI scans, No. (%)	951 (100)	687 (72)	264 (28)
No. of MRI scans per participant, median (IQR)	5 (4-6)	5 (4-7)	5 (4-5)
Age at baseline MRI scans, y			
Mean (SD)	55.4 (8.4)	53.1 (7.8)	57.2 (8.9)^a^
Median (IQR)	55 (52-60)	53 (50-59)	57 (54-62)^a^
Sex, No. (%)			
Men	69 (37)	45 (36)	24 (40)
Women	116 (63)	80 (64)	36 (60)
Duration of follow-up, y			
Mean (SD)	20.2 (3.0)	20.6 (3.2)	19.7 (2.9)
Median (IQR)	20 (18-22)	21 (19-23)	20 (18-21)
Educational attainment, y			
Mean (SD)	17.3 (2.3)	17.6 (2.6)	17.1 (2.2)
Median (IQR)	18 (16-20)	18 (17-20)	17 (16-19)
Vascular risk factors, No. (%)			
Hypertension	51 (28)	31 (25)	20 (33)
Dyslipidemia	57 (31)	34 (27)	23 (38)
Type 2 diabetes	19 (10)	8 (6)	11 (18)^b^
Smoking	6 (3)	4 (3)	2 (3)
No. of vascular risks per participant, No. (%)			
0	91 (49)	66 (53)	25 (42)
1	64 (35)	43 (34)	21 (35)
2	23 (12)	14 (11)	9 (15)
3	5 (3)	2 (2)	3 (5)
4	2 (1)	0	2 (3)
CSF biomarkers at baseline, No. (%)			
Low Aβ_42_:Aβ_40_ ratio	17 (9)	7 (6)	10 (17)^b^
Positive for p-tau181	6 (3)	2 (2)	4 (7)
Positive for t-tau	8 (4)	3 (2)	5 (8)
*APOE* ε4 allele, No. (%)	60 (32)	36 (29)	24 (40)

Abbreviations: Aβ_40_, amyloid-β peptide 40; Aβ_42_, amyloid-β peptide 42; CSF, cerebrospinal fluid; MCI, mild cognitive impairment; MRI, magnetic resonance imaging; p-tau181, tau phosphorylated at threonine 181; t-tau, total tau.

^a^ *P* < .01 between the individuals with continued normal cognition and those with progression to MCI.

^b^ *P* < .05 between the individuals with continued normal cognition and those with progression to MCI.

### Longitudinal Changes in Brain Volumes

**Figure 1.**
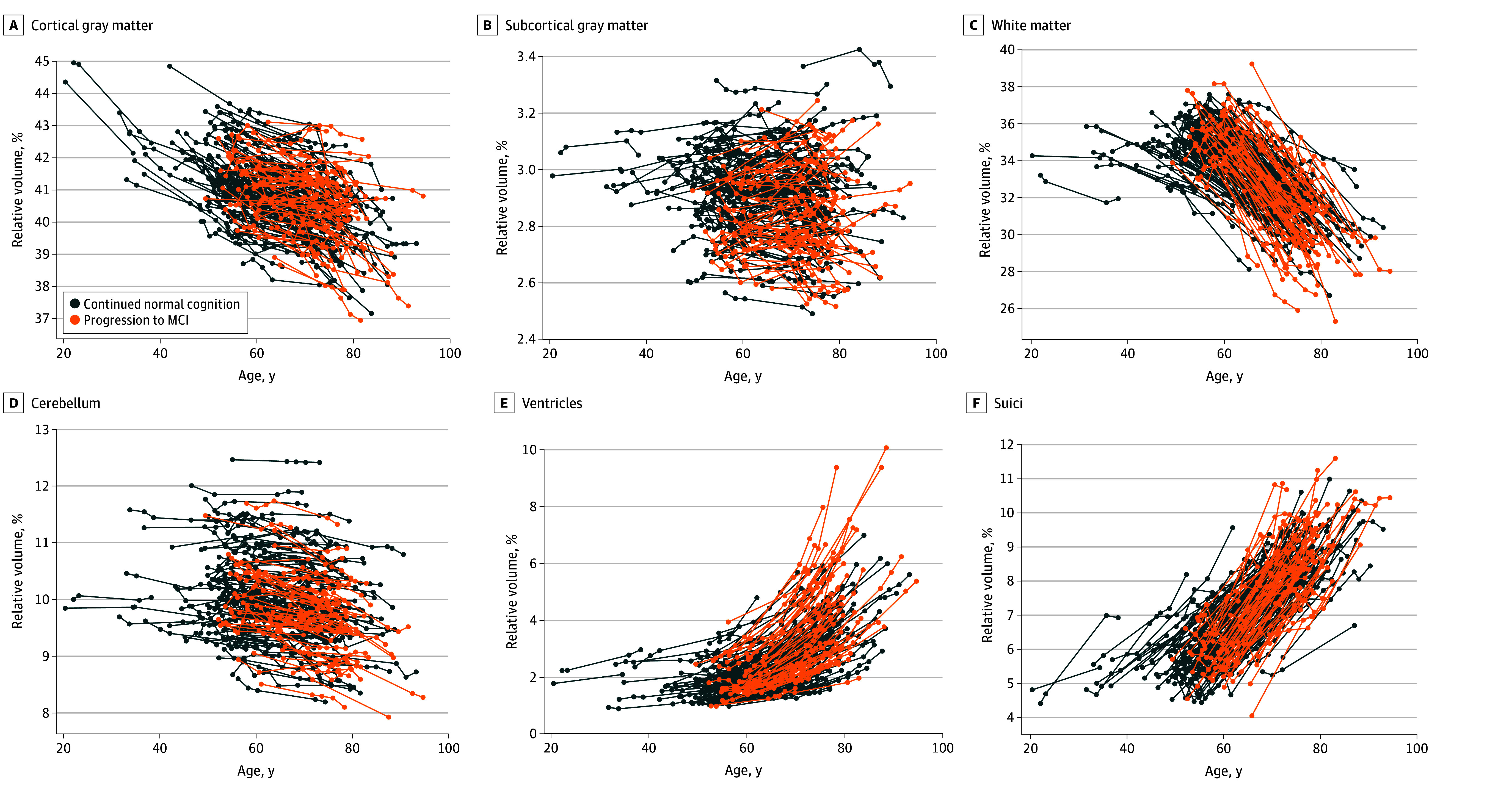
Longitudinal Changes in Brain Volumes Across Ages Intracranial volume–adjusted longitudinal volumes of brain structures are plotted as a function of participants’ age. Each line indicates 15 to 27 years of observations for an individual. MCI indicates mild cognitive impairement.

Intracranial volume-adjusted longitudinal brain volumes (ie, relative brain volumes) are shown as a function of participants’ age in Figure 1. The relative brain volumes as a function of years since baseline are also shown in eFigure 3 in Supplement 1. Overall, the relative volumes of the cortical gray matter and white matter decreased over time, whereas those of the ventricles and sulci increased over time.

### Annual Change Rates of Brain Volumes

**Figure 2.**
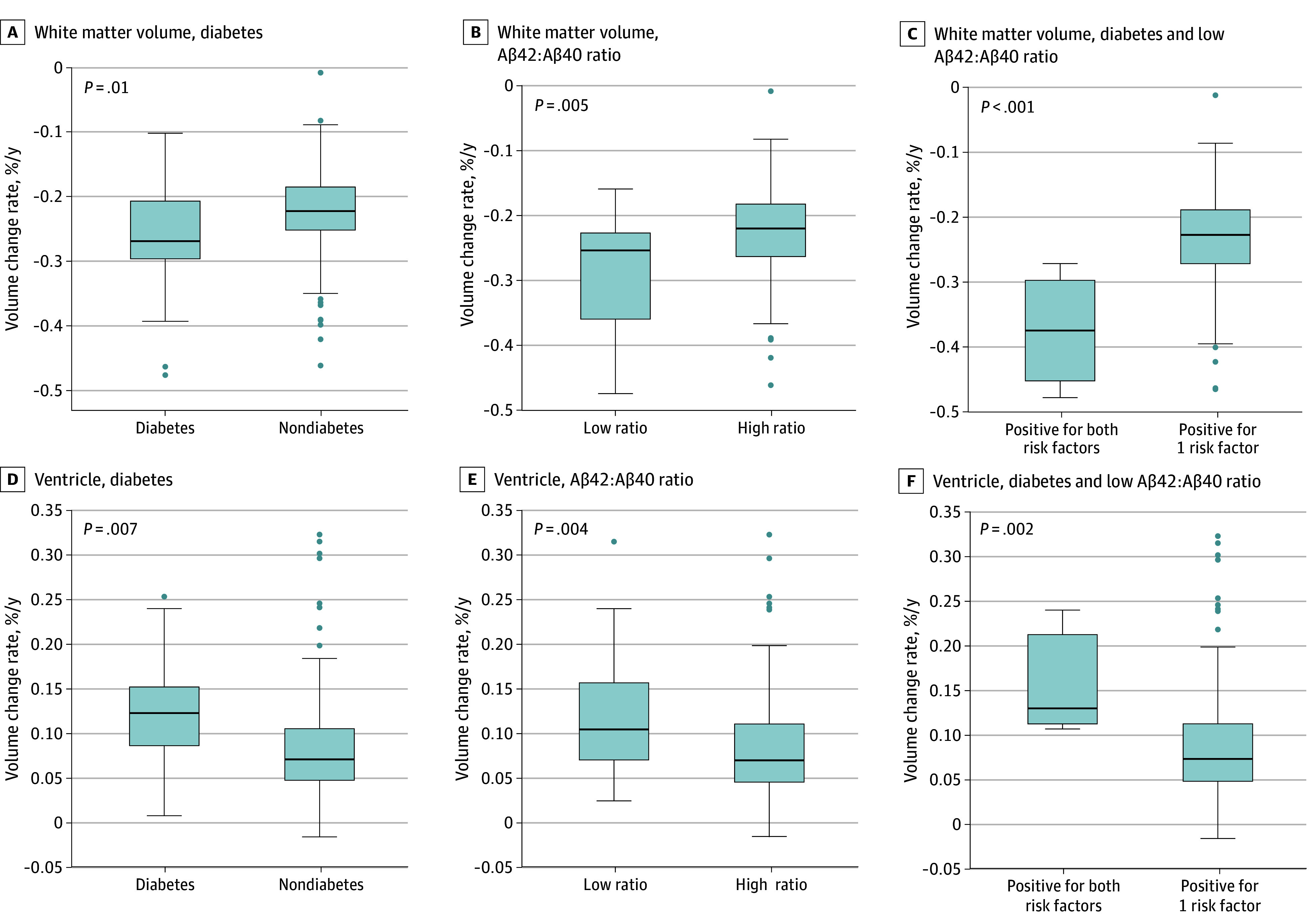
Annual Change Rates in Brain Volumes Includes changes in white matter atrophy and ventricular enlargement. Annual rates of change in brain volumes were stratified by the presence of diabetes, positivity for a low cerebrospinal fluid (CSF) ratio of amyloid β peptide 42 [Aβ_42_] to Aβ_40_ (a low ratio is positive and a high ratio is negative), and both risk factors. Boxes indicate IQR; error bars, range; horizontal lines, median; and data points, outliers (beyond 1.5 × IQR from either end of the box).

The relative volumes of the cortical gray matter and white matter demonstrated mean (SD) annual decreases of 0.0053% (0.0034%) and 0.0229% (0.0067%), respectively, whereas those of the ventricles and sulci showed mean (SD) annual increases of 0.0089% (0.0058%) and 0.0144% (0.0036%), respectively. Among the vascular risk factors and CSF biomarkers, significant differences in the annual rates of change were found between participants with vs without diabetes in the white matter (mean difference, 0.0439% [95% CI, 0.0078%-0.0798]; *P* = .01) and the ventricles (mean difference, 0.0395% [95% CI, 0.0142%-0.0648%]; *P* = .007]) (Figure 2) and between participants with a high vs low Aβ_42_:Aβ_40_ ratio in the white matter (mean difference, 0.0638% [95% CI, 0.0153%-0.1123%]; *P* = .005) and the ventricles (mean difference, 0.0421% [95% CI, 0.0031%-0.0812%]; *P* = .004). These differences were most prominent in groups with both diabetes and a low Aβ_42_:Aβ_40_ ratio compared with groups who had one but not both risk factors for the white matter (mean difference, 0.1434% [95% CI, 0.0248%-0.2621%]; *P* < .001) and the ventricles (mean difference, 0.0706% [95% CI, 0.0106%-0.1319%]; *P* = .002). Meanwhile, there were no significant differences in the annual rates of change between participants with vs without other vascular risk factors (hypertension, dyslipidemia, smoking), positivity for other CSF biomarkers (p-tau181 and t-tau), and *APOE* ε4 status (eFigure 4 in Supplement 1).

Histograms showing the frequency of annual rates of change of the relative brain volumes among study participants are shown in eFigure 5 in Supplement 1. Change-point analyses detected the age of 62.4 years in the white matter and 68.9 years in the ventricle, indicating that the white matter atrophy and ventricular enlargement accelerated at these ages (eFigure 6 in Supplement 1). No change points of the age were detected in the slopes of the other brain structures.

### Survival Analyses

**Figure 3.**
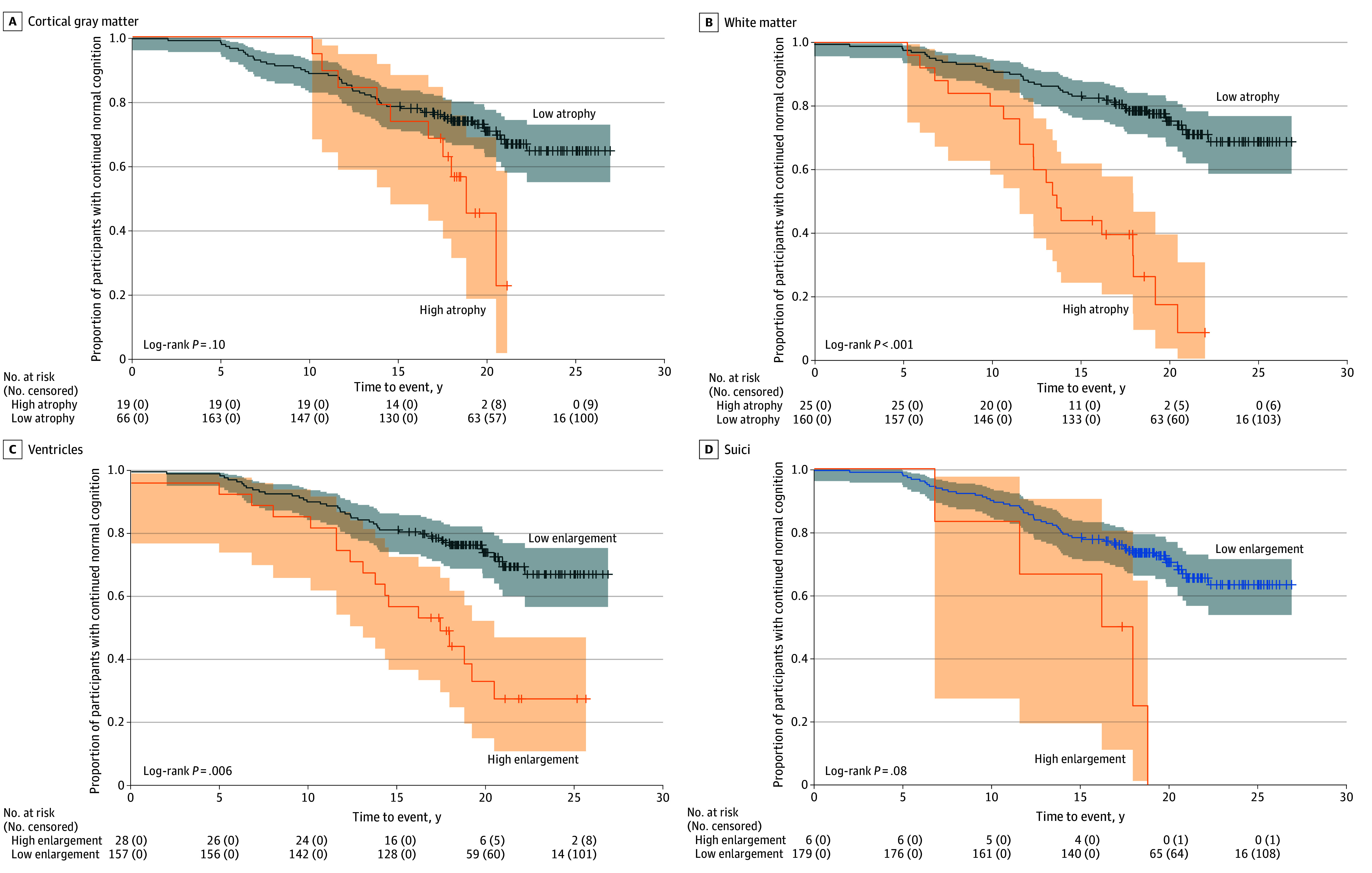
Kaplan-Meier Survival Plots With Log-Rank Test The Kaplan-Meier survival curves are separately displayed for the groups with high and low levels of atrophy of the cortical gray matter and white matter and for those with high and low levels of enlargement of the ventricles and sulci. Shaded areas indicate 95% CIs for each group.

The Kaplan-Meier survival curves visualized the estimated probability of progression from normal cognition to MCI for participants with high vs low rates of atrophy and enlargement of the regional brain volumes (Figure 3). The log-rank tests in the white matter and ventricles demonstrated earlier progression from normal cognition to MCI symptom onset in the groups with high levels of atrophy and enlargement compared with those in the groups with low levels of atrophy and enlargement. Additionally, the presence of diabetes (χ^2^ = 3.99; *df*, 1; log-rank *P* = .046) and low CSF Aβ_42_:Aβ_40_ ratio (χ^2^ = 4.10; *df*, 1; log-rank *P* = .04) were associated with progression from normal cognition to MCI symptom onset. Participants with both diabetes and a low CSF Aβ_42_:Aβ_40_ ratio demonstrated a higher rate of MCI progression (χ^2^ = 4.77; *df*, 1; log-rank *P* = .03) (eFigure 7 in Supplement 1) than participants with one but not both risk factors.

The HRs of variables determined by the multivariable Cox proportional hazards regression model are listed in Table 2. High rates of volume change were associated with the progression from normal cognition to MCI symptom onset in the white matter (HR, 1.86 [95% CI, 1.24-2.49]; *P* = .001) and the ventricles (HR, 1.71 [95% CI, 1.19-2.24]; *P* = .009). Age (HR, 1.06 [95% CI, 1.03-1.10]; *P* < .001), the presence of type 2 diabetes (HR, 1.41 [95% CI, 1.06-1.76]; *P* = .04), and low CSF Aβ_42_:Aβ_40_ ratio (HR, 1.48 [95% CI, 1.09-1.88]; *P* = .04) were also associated with progression to MCI. Furthermore, the combination of diabetes and low CSF Aβ_42_:Aβ_40_ ratio had a higher HR than each risk factor separately (HR, 1.55 [95% CI, 1.13-1.98]; *P* = .03), indicating a synergic association of diabetes and amyloid pathology with MCI progression.

**Table 2.** Hazard Ratios for Estimation of Progression to MCI^a^

Variable	HR (95% CI)	*P* value
Demographic characteristics		
Age	1.06 (1.03-1.10)	<.001
Women	0.89 (0.73-1.17)	.60
Educational level	0.95 (0.86-1.04)	.37
High rates of change in volume		
Cortical gray matter	1.19 (0.88-1.51)	.11
Subcortical gray matter	0.97 (0.71-1.23)	.81
White matter	1.86 (1.24-2.49)	.001
Cerebellum	1.10 (0.92-1.29)	.21
Ventricles	1.71 (1.19-2.24)	.009
Sulci	1.33 (0.93-1.74)	.07
Vascular risk factors		
Hypertension	1.31 (0.85-1.78)	.11
Dyslipidemia	1.25 (0.83-1.66)	.16
Diabetes	1.41 (1.06-1.76)	.04
Smoking	1.22 (0.64-1.79)	.28
Abnormal CSF measures		
Low Aβ_42_:Aβ_40_ ratio	1.48 (1.09-1.88)	.04
Positive for p-tau181	1.25 (0.79-1.70)	.09
Positive for t-tau	1.27 (0.82-1.75)	.08
*APOE* ε4 status	1.30 (0.89-1.72)	.08
Diabetes × Aβ_42_:Aβ_40_ ratio	1.55 (1.13-1.98)	.03

Abbreviations: Aβ_40_, amyloid-β peptide 40; Aβ_42_, amyloid-β peptide 42; CSF, cerebrospinal fluid; HR, hazard ratio; MCI, mild cognitive impairment; p-tau181, tau phosphorylated at threonine 181; t-tau, total tau.

^a^ Data are derived from a multivariable Cox proportional hazards regression model.

## Discussion

In this cohort study, we analyzed the longitudinal changes in the whole brain volumes, segmented into the cortical gray matter, subcortical gray matter, whole-brain white matter, cerebellum, total ventricles, and whole-brain sulci among middle-aged and older participants with normal cognition at baseline, including approximately one-third of participants with progression to MCI during the mean follow-up period of 20.2 years. This analysis characterized the rates of change in anatomical brain volumes over time and their associations with vascular risk factors and CSF biomarkers for Alzheimer disease pathology, as well as the abilities of these measures to estimate progression from normal cognition to MCI.

Our long-term longitudinal cohort study demonstrated considerable interindividual and intraindividual variations in annual rates of change in the whole-brain white matter volumes among individuals with unimpaired cognition at baseline. This intraindividual variability is challenging to elucidate in cross-sectional studies for the brain structural alterations and rates of change over the human lifespan,^1,31^ where the overall peak of the white matter volume was at about 40 years of age and then declined. In line with this, some longitudinal studies^32,33^ have shown that degeneration of the white matter started around 40 years of age and progressed with age-associated acceleration. In addition to the results of these previous studies, the present longitudinal study demonstrated that individuals with higher change rates in the white matter volumes were more likely to develop onset of MCI symptoms over time. These findings highlight that white matter volume changes are closely associated with cognitive function in aging, suggesting that white matter degeneration may play a crucial role in cognitive decline.

Importantly, diabetes, low CSF Aβ_42_:Aβ_40_ ratio, and their combination were associated with higher rates of change in the white matter atrophy and ventricular enlargement over time. Individuals with both risk factors were more prone to MCI progression than individuals with one but not both. Previous longitudinal brain MRI studies have not reported the synergic association of diabetes and Alzheimer disease pathology with rates of change in brain volume but demonstrated the association of diabetes with brain volume loss, particularly reflected by the accelerated expansion of the ventricle, indicating that brain atrophy may be most pronounced in the white matter regions surrounding the ventricles.^20,34,35^ Similarly, individuals with lower Aβ_42_:Aβ_40_ ratios had increased white matter lesions and enlarged lateral ventricles.^36,37^ As insulin resistance plays a critical role in the formation of amyloid plaques,^38^ diabetes may promote Alzheimer disease pathology, resulting in an earlier progression from normal cognition to MCI.^24,39^ These results suggest that controlling diabetes may help reduce the risk of Alzheimer dementia later in life as a modifiable risk factor.^40^

In contrast to diabetes and low Aβ_42_:Aβ_40_ ratio, other vascular risk factors (hypertension, dyslipidemia, smoking) and CSF biomarkers (p-tau181 and t-tau) were not associated with annual change rates of the brain volumes in the present study. These results were unexpected, given previous studies using the BIOCARD cohort, which found that these vascular risk factors and CSF biomarkers were linked with subsequent cognitive decline.^41,42,43^ This could be attributable to insufficient effect sizes for these vascular risk factors and sample sizes for the p-tau181– and t-tau–positive groups. The effect of diabetes on brain structure volumes has previously been reported to be greater than that of hypertension and dyslipidemia.^17^ Consistent with CSF p-tau181 and t-tau values being closely associated with progression in Alzheimer disease symptoms,^44^ few individuals with normal cognition had positive values for these CSF biomarkers at baseline. In addressing these discrepancies, a future longitudinal study would be warranted to design a larger sample size including individuals with MCI at baseline, especially for subgroups with positivity for p-tau181 and t-tau.

### Limitations

The present study has several limitations. First, the sample size of 185 participants was relatively small and the sex proportion was imbalanced in the present study. Although previous studies demonstrated sex differences in brain structures and functions,^45,46^ it was challenging to conduct a meaningful sexwise analysis. Second, serial MRI scan parameters for participants in the BIOCARD cohort were changed, accompanied by the scan site transition from the NIH to JHU and the availability of 3T image acquisition. Even after applying a longitudinal method for the data harmonization,^28^ the method of brain volume measurements using a deep learning–based method could underestimate intracranial volumes,^27^ leading to an apparent increase in some regions. Third, the individuals involved in the BIOCARD cohort were predominantly well-educated and White, with most having a family history of dementia, which suggests that the results might not be generalizable to the broader population.

## Conclusions

In this cohort study of middle-aged and older adults with normal cognition at recruitment and a mean follow-up of 20.2 years, we analyzed the longitudinal data of volumetric MRI, CSF, and clinical and cognitive assessments. Higher rates of change in the white matter and ventricle volumes, along with the presence of diabetes and low CSF Aβ_42_:Aβ_40_ ratio, were identified as significant risk factors for the progression to MCI. These results support the importance of identifying individuals who have accelerated brain atrophy to optimize preventive strategies for progression to MCI.

## Notes

Supplement 1. **eMethods.** Study Participants and Assessments **eFigure 1.** Coronal, Sagittal, and Ensemble SPGR Images **eFigure 2.** Multiple Hierarchical Structural Levels **eFigure 3.** Longitudinal Changes in Brain Segmental Volumes Across Years **eFigure 4.** Volume Change Rates Among Vascular Risk Factors, CSF Biomarkers, and Genetics **eFigure 5.** Histograms of Annual Rate Changes of Brain Volumes **eFigure 6.** Change-Point Analyses in the White Matter and Ventricles **eFigure 7.** Kaplan-Meier Survival Curves With Log-Rank Test **eTable.** Number of MRI Scans in Each Year
eReferences.

Supplement 2.
Data Sharing Statement
