# Supplementary material for: Acceleration of Brain Atrophy and Progression From Normal Cognition to Mild Cognitive Impairment
Source: JAMA Netw Open. 2024 Oct 30;7(10):e2441505. doi: 10.1001/jamanetworkopen.2024.41505 (PMC11525609; doi:10.1001/jamanetworkopen.2024.41505)
Supplement: Supplement 1. — eMethods. Study Participants and Assessments eFigure 1. Coronal, Sagittal, and Ensemble SPGR Images eFigure 2. Multiple Hierarchical Structural Levels eFigure 3. Longitudinal Changes in Brain Segmental Volumes Across Years eFigure 4. Volume Change Rates Among Vascular Risk Factors, CSF Biomarkers, and Genetics eFigure 5. Histograms of Annual Rate Changes of Brain Volumes eFigure 6. Change-Point Analyses in the White Matter and Ventricles eFigure 7. Kaplan-Meier Survival Curves With Log-Rank Test eTable. Number of MRI Scans in Each Year eReferences. [file jamanetwopen-e2441505-s001.pdf]

Supplementary Online Content

Uchida Y, Nishimaki K, Soldan A, Moghekar A, Albert M, Oishi K. Acceleration of brain atrophy and progression from normal cognition to mild cognitive impairment. *JAMA Netw Open*. 2023;6(10):e2441505.
doi:10.1001/jamanetworkopen.2024.41505

eMethods. Study Participants and Assessments

eFigure 1. Coronal, Sagittal, and Ensemble SPGR Images

eFigure 2. Multiple Hierarchical Structural Levels

eFigure 3. Longitudinal Changes in Brain Segmental Volumes Across Years

eFigure 4. Volume Change Rates Among Vascular Risk Factors, CSF Biomarkers, and Genetics

eFigure 5. Histograms of Annual Rate Changes of Brain Volumes

eFigure 6. Change-Point Analyses in the White Matter and Ventricles

eFigure 7. Kaplan–Meier Survival Curves With Log-Rank Test

eTable. Number of MRI Scans in Each Year

eReferences.

This supplemental material has been provided by the authors to give readers additional information about their work.

©2023 Uchida Y et al. *JAMA Network Open*.

eMethods. Study Participants and Assessments

The BIOCARD cohort

The BIOCARD cohort, initiated at the National Institutes of Health in 1995, was established with the aim of identifying predictive variables among cognitively normal individuals that might indicate the eventual onset of Alzheimer's disease.[1] Participants, approximately three-quarters of whom had a family history of dementia according to the cohort design, underwent neuropsychological batteries and clinical assessments annually. In addition, their MRI scans and CSF specimens were obtained every 2 years. The cohort study was suspended in 2005 for administrative reasons and re-established at Johns Hopkins University in 2009. The bi-annual collection of MRI and CSF was re-initiated in 2015. To date, more than 400 participants have been enrolled in this cohort across 28 years.[2]

Participants in the present study

Each participant in this study was given a consensus diagnosis annually by the Clinical Core of the BIOCARD study team at JHU, based on a comprehensive assessment.[1] On an annual basis, participants were administered a neuropsychological battery, the Clinical Dementia Rating (CDR) scale, which consists of a semi-structured interview with the participant and study partner, and a medical evaluation that included neurological and psychiatric examinations. The diagnostic criteria adhered to the recommendations of the National Institute on Aging and the Alzheimer's Association working groups for diagnosing MCI[3] and dementia.[4] The diagnosis for each participant was established by evaluating longitudinal cognitive performance across all major cognitive domains, clinical information regarding participants' medical, neurological, and psychiatric status, and reports of cognitive change by the participants and study partner during the CDR interview. Participants gave written informed consent, and the study was approved by the Johns Hopkins Medicine Institutional Review Board.

Neuropsychological assessment

All of the neuropsychological assessments conducted by the Johns Hopkins research team were also conducted during participants' evaluations at the National Institutes of Health, with one exception as described below. The assessment battery encompassed a wide range of cognitive domains, including memory, executive function, language, visuospatial ability,

©2023 Uchida Y et al. *JAMA Network Open*.

attention, speed of processing, and psychomotor speed. The examination battery comprised the following assessments: the Logical Memory (raw score and percent Retention) and Paired Associates Subtests of the Wechsler Memory Scale-Revised, the California Verbal Learning Test; the Rey-Osterreith Complex Figure (copy and recall); the Trail Making Test, Parts A and B; the Boston Naming Test (30-item version); the Letter and Category Fluency; the Block Design subtest of the Wechsler Adult Intelligence Scale-Revised; the Digit Span Forward and Backward; the Digit Symbol Test; the Mini-Mental State Examination; and the Lafayette Grooved Pegboard. The latter was the only assessment included in the battery at Johns Hopkins University. At the National Institutes of Health, additional neuropsychological tests were conducted: the complete Wechsler Adult Intelligence Scale-Revised, the full Wechsler Memory Scale-Revised; a variant of the Buschke Cued Selective Reminding Test; the Stroop Test; the Delis-Kaplan Executive Function System; and Clock Drawing.

Vascular risk assessments

For hypertension, dyslipidemia, and diabetes, absence was defined as never having a formal diagnosis, whereas smoking was defined as currently or within the last 30 days. The participants who had at least one presence of these vascular risk factors during any of their visits were counted as having that risk factor. A summary vascular risk score was calculated by summing these dichotomous variables (each coded as 0 = absence vs. 1 = presence).

- Definition at baseline evaluation

All vascular risk factors, including hypertension, dyslipidemia, diabetes, and smoking, were initially defined based on the baseline evaluation. These were determined through self-reported medical history interviews and medical records at the time of the first visit. The presence or absence of these factors was established at the baseline.

- Handling changes during the follow-up period

If any of these vascular risk factors changed during the follow-up period, such as a new diagnosis or resumption of smoking, these changes were recorded during subsequent visits. In calculating the overall vascular risk score throughout the study, we considered the presence of each risk factor at any point during the follow-up period. In other words, if a participant had any risk factor at any time during the study, it was counted as a positive risk factor in their scores.

©2023 Uchida Y et al. *JAMA Network Open*.

- Consideration of treatments

When a participant received a vascular risk factor treatment during the follow-up period, we recorded whether the risk factor was still present after treatment. Even if hypertension was managed through treatment, participants with a history of hypertension were still counted as having that risk factor. This approach ensured that the study results appropriately accounted for the influence of risk factor management through treatment.

MRI acquisition for brain volume analysis

Scans using a GE 1.5T scanner acquired at the National Institutes of Health from 1995 to 2005 included the SPGR sequence with a coronal slice orientation (repetition time = 24 ms, echo time = 2 ms, flip angle = 20°, field of view = 240 × 248 × 240 mm^3, matrix size = 256 × 256 mm^2, and slice thickness = 2 mm, 124 slices with 0.9375 × 2 × 0.9375 mm^3 resolution) and the SPGR sequence with a sagittal slice orientation (repetition time = 24 ms, echo time = 3 ms, flip angle = 45°, field of view = 240 × 240 × 186 mm^3, matrix size = 256 × 256 mm^2, and slice thickness = 1.5 mm, 124 slices with 0.9375 × 0.9375 × 2 mm^3 resolution).

Scans using a Philips 3T scanner acquired at Johns Hopkins University from 2015 to 2023 included the MPRAGE sequence with a sagittal slice orientation (repetition time = 6.7 ms, echo time = 3.1 ms, flip angle = 8°, inversion time = 843 ms, shot interval = 3000 ms, field of view = 256 × 256 × 204 mm^3, matrix size = 256 × 256 mm^2, and slice thickness = 1.2 mm, 170 slices with 1 × 1 × 1.2 mm^3 resolution).

Brain parcellation and volume measurement

Whole-brain parcellation was performed on the three-dimensional T1-weighted images using a fully automated deep learning method, known as Open-source Multiple Anatomical Parcellation T1 (OpenMAP-T1),[5] which is freely accessible through the website (URL: https://github.com/OishiLab/OpenMAP-T1). This tool can parcellate whole-brain T1-weighted images into 280 anatomical regions based on the JHU-MNI atlas. The accuracy of brain parcellation has been demonstrated to be robust across various scanner types, magnetic field strengths, and scan protocols, such as the SPGR and MPRAGE sequences.[5] Before the parcellation of the SPGR images, the coronal and sagittal SPGR images were realigned and combined, yielding the ensemble SPGR images with isotropic voxel resolutions (eFigure 1 in Supplement 1). After processing these images through OpenMAP-T1, the parcellated 280

©2023 Uchida Y et al. *JAMA Network Open*.

anatomical regions were integrated into multiple hierarchical structural levels.[6] Each level contained a set of predefined regions of interest,[7] and volumes of the following segmental brain areas were calculated and included in the analyses reported below: the cortical gray matter; subcortical gray matter; whole-brain white matter; cerebellum; total ventricles; and whole-brain sulci (eFigure 2 in Supplement 1). To allow for comparisons among individuals with differing head sizes, the raw volume of each brain structure was adjusted for head size by dividing the raw volume by the intracranial volume of each individual, which was determined by summing the volumes of all 280 anatomical regions at each scan.

CSF assessments

CSF samples were obtained via lumbar puncture and aliquoted into low-bind polypropylene cryotubes, which were placed on dry ice, and transferred to a freezer set at −80◦C for long term storage. For the present analysis, the samples were thawed for the first time to undergo fully automated chemiluminescence enzyme immunoassays using a Lumipulse G1200 system (FujirebioDiagnostics, Inc.). The concentration for Aβ40, Aβ42, tau phosphorylated at threonine 181 (p-tau181), and total tau (t-tau) was measured. The Aβ42/Aβ40 ratio was used as an index for amyloid pathology to account for inter-individual differences in total CSF Aβ levels,[8] whereas p-tau181 and t-tau were used to assess the extent of tau pathology and neurodegeneration, respectively.[9] Participants in the present study were divided into the biomarker positive and negative groups at baseline using the cut-off values for positivity of each CSF biomarker, based on a prior Lumipuse CSF assay: Aβ42/Aβ40 < 0.069; p-tau181 > 56.5 pg/ml; and t-tau > 400 pg/ml.[10]

APOE Genotype

APOE genotypes were determined using the restriction endonuclease digestion of a polymerase chain reaction amplified genomic DNA system (Athena Diagnostics, Inc.). An APOE ε4 carrier status was assigned as follows: individuals carrying at least one ε4 allele were coded as 1, while noncarriers were coded as 0.

©2023 Uchida Y et al. *JAMA Network Open*.

eFigure 1. Coronal, Sagittal, and Ensemble SPGR Images

eFigure 1. Coronal, sagittal, and ensemble SPGR images

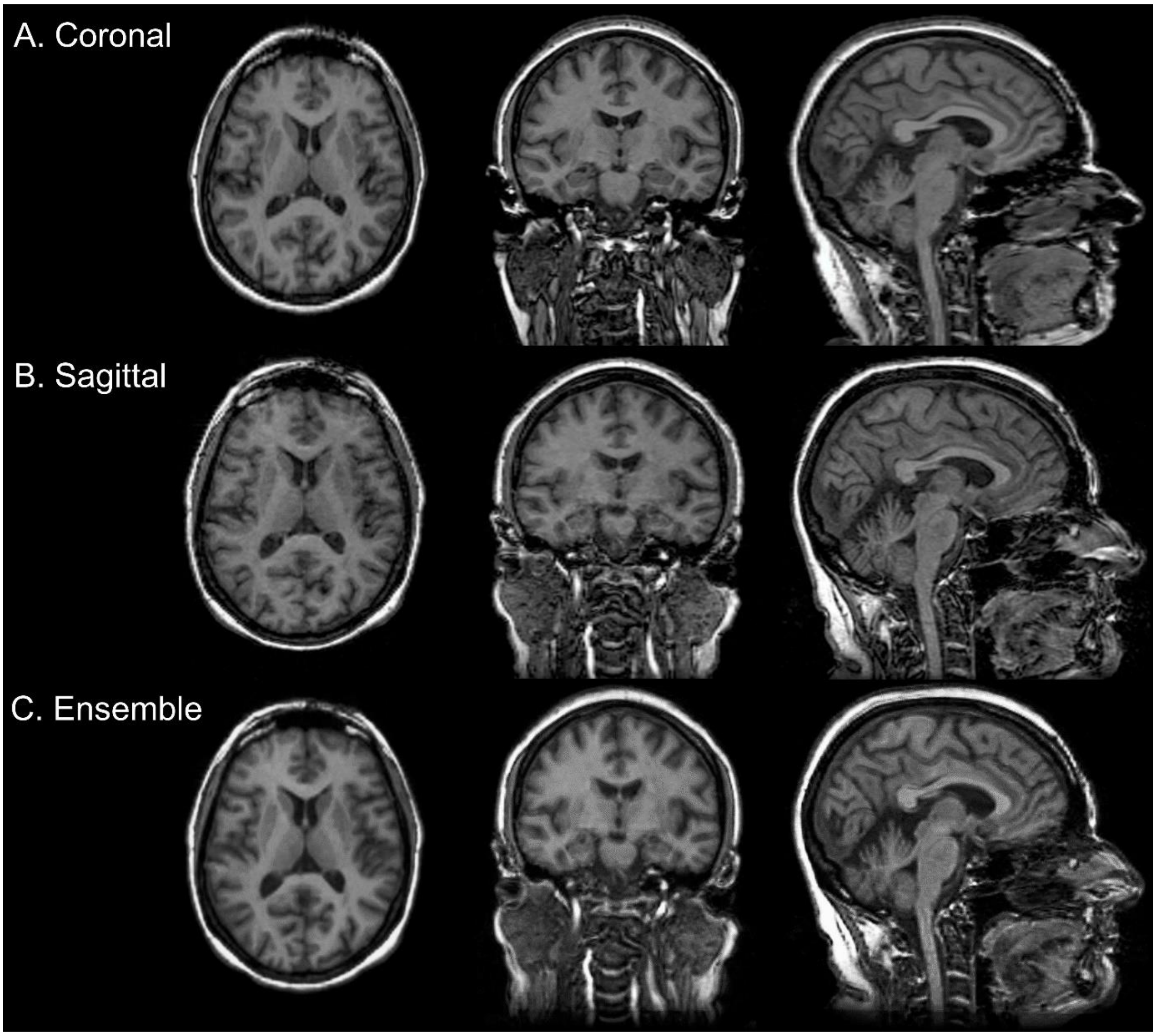

(A) Coronal SPGR: 0.9375 × 2 × 0.9375 mm^3 resolution. (B) Sagittal SPGR: 0.9375 × 0.9375 × 2 mm^3 resolution. (C) Ensemble SPGR: 0.9375 × 0.9375 × 0.9375 mm^3 resolution. Note that the signal-to-noise ratio of ensemble SPGR is improved in each direction.

©2023 Uchida Y et al. *JAMA Network Open*.

eFigure 2. Multiple Hierarchical Structural Levels

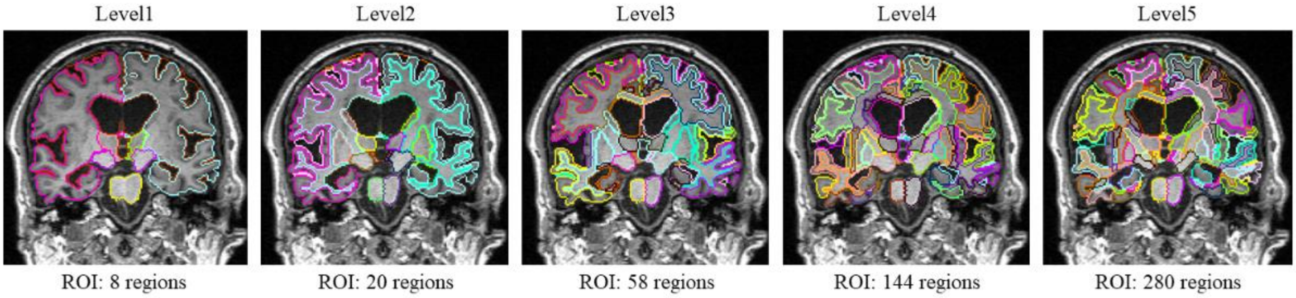

(https://github.com/OishiLab/OpenMAP-T1)

OpenMAP-T1 parcellates a whole brain into five hierarchical structural levels (Level 1: Hemispheric level; Level 2: Gray and white matter segmentation level; Level 3: Lobar level; Level 4: Coarse parcellation level; Level 5: Fine parcellation level).

©2023 Uchida Y et al. *JAMA Network Open*.

eFigure 3. Longitudinal Changes in Brain Volumes Across Years

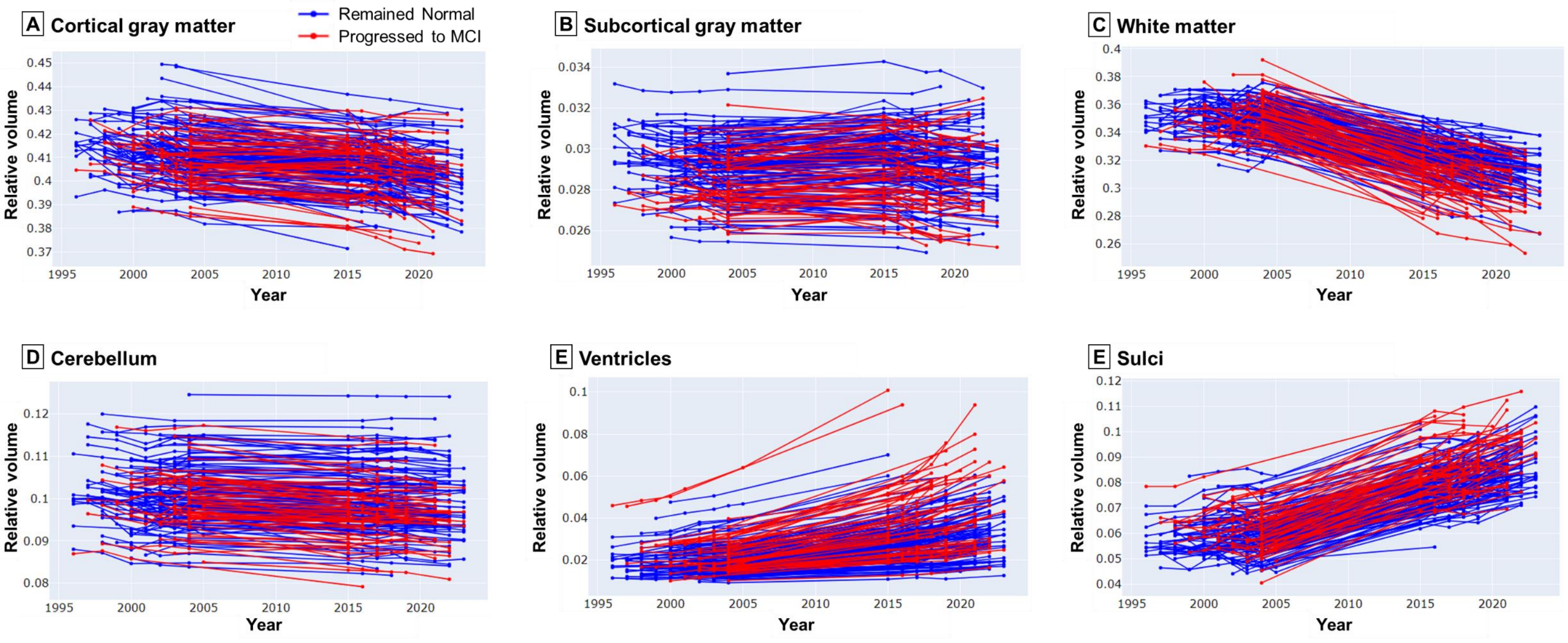

©2023 Uchida Y et al. *JAMA Network Open*.

Intracranial volume-adjusted longitudinal volumes of brain structures are plotted as a function of years. Each line indicates 15 to 27 years of observations for an individual. Blue colored lines are individuals who remained with normal cognition and red colored lines are individuals who progressed to MCI.

©2023 Uchida Y et al. *JAMA Network Open*.

eFigure 4. Volume Change Rates Among Vascular Risk Factors, CSF Biomarkers, and Genetics

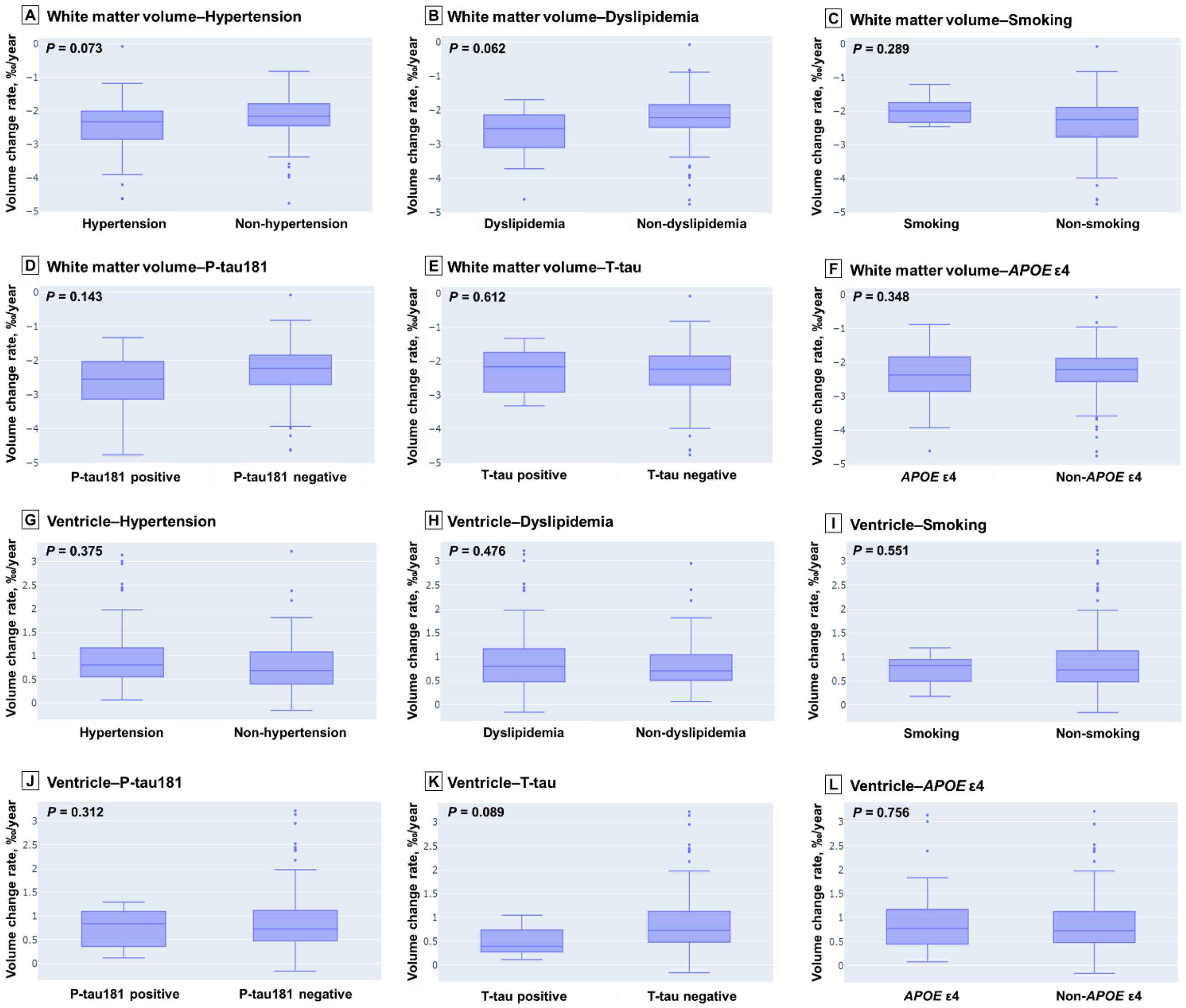

No significant differences in the white matter and ventricle volume change rates were found among the vascular risk factors (hypertension, dyslipidemia, and smoking), CSF biomarkers (p-tau181 and t-tau), and genetics (*APOE* ε4 status).

©2023 Uchida Y et al. *JAMA Network Open*.

eFigure 5. Histograms of Annual Change Rates of Brain Volumes

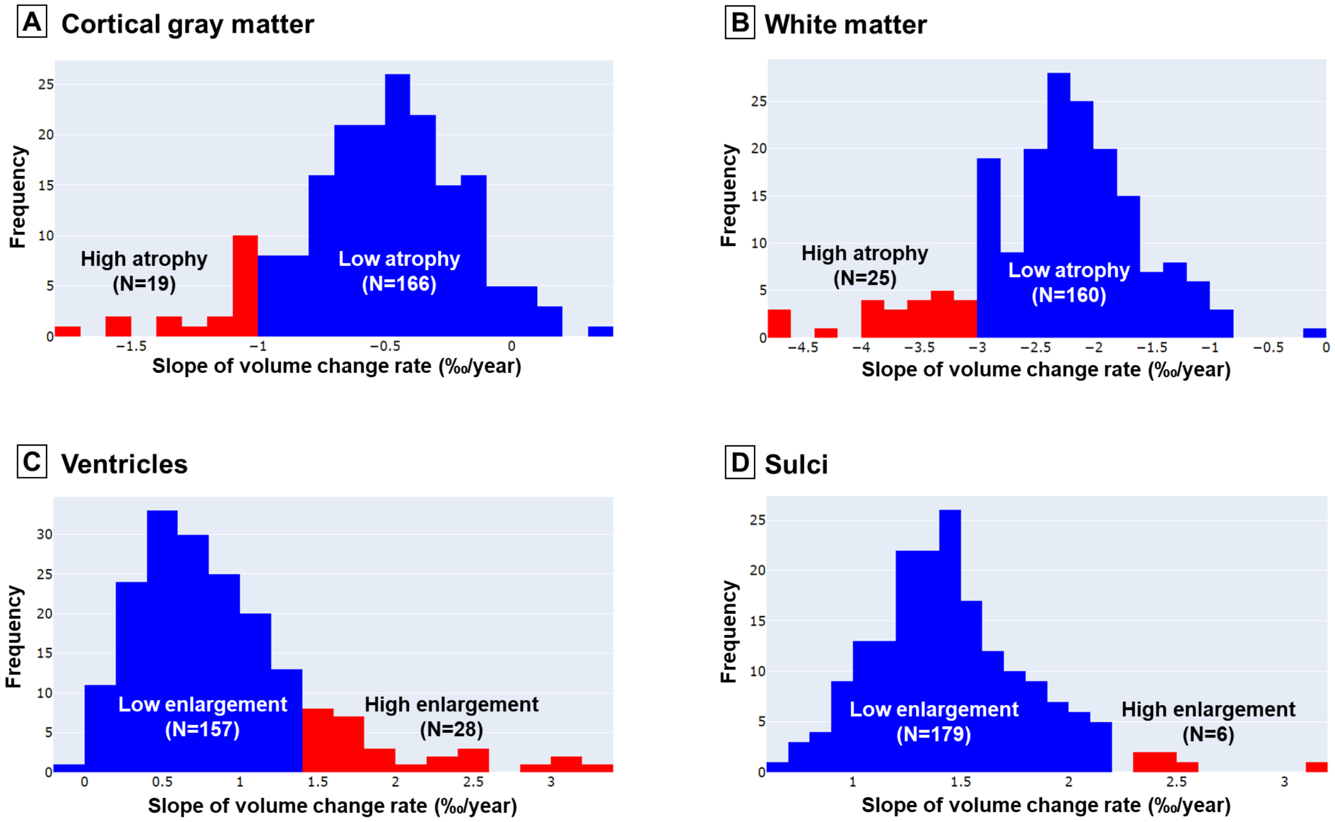

Individuals were divided into the high/low atrophy and the high/low enlargement groups according to the Gaussian mixture models for the slopes of the annual change rate of the brain volumes. Note that the high group is colored in red and the low group is in blue.

©2023 Uchida Y et al. *JAMA Network Open*.

eFigure 6. Change-Point Analyses in the White Matter and Ventricles

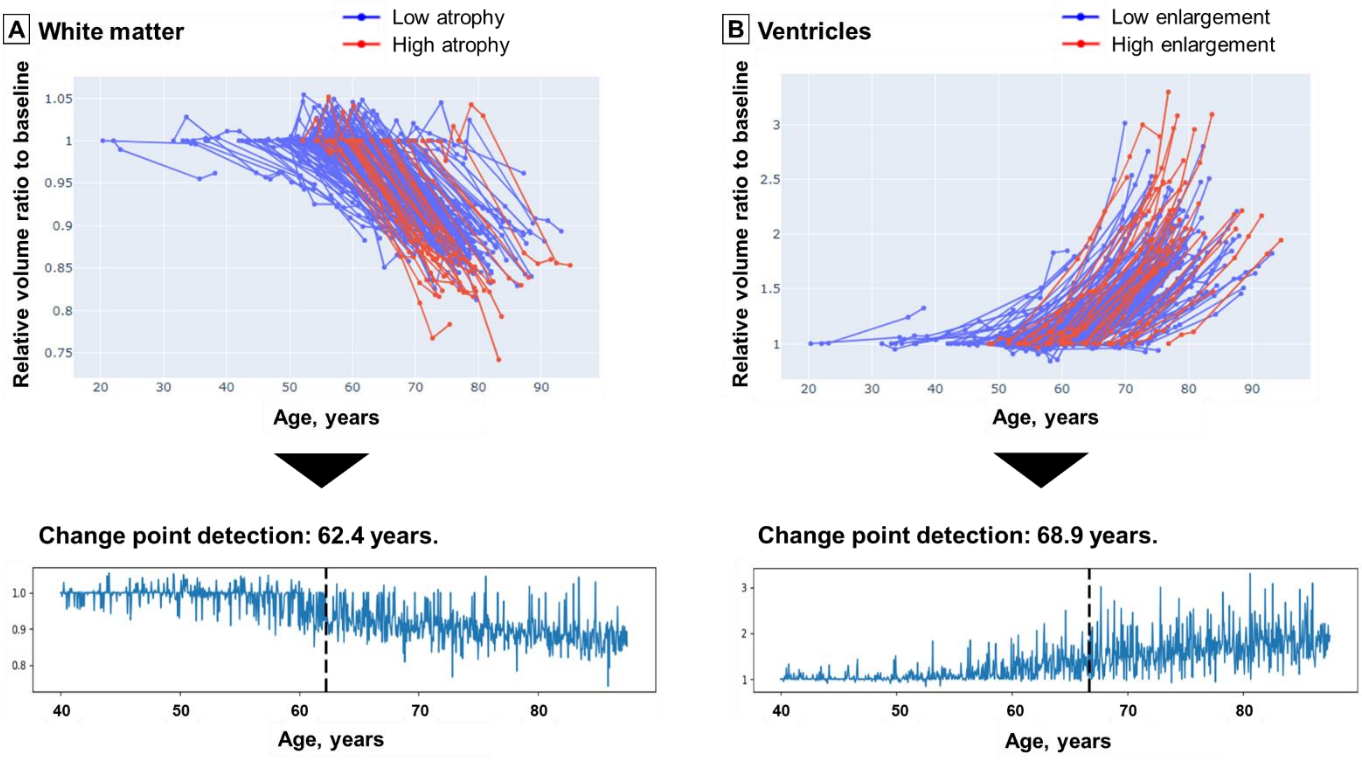

Change-point analyses detected the age of 62.4 years in the white matter and 68.9 years in the ventricle, indicating that the white matter atrophy and ventricular enlargement were accelerated at these ages.

©2023 Uchida Y et al. *JAMA Network Open*.

eFigure 7. Kaplan-Meier Survival Curves With Log-Rank Test

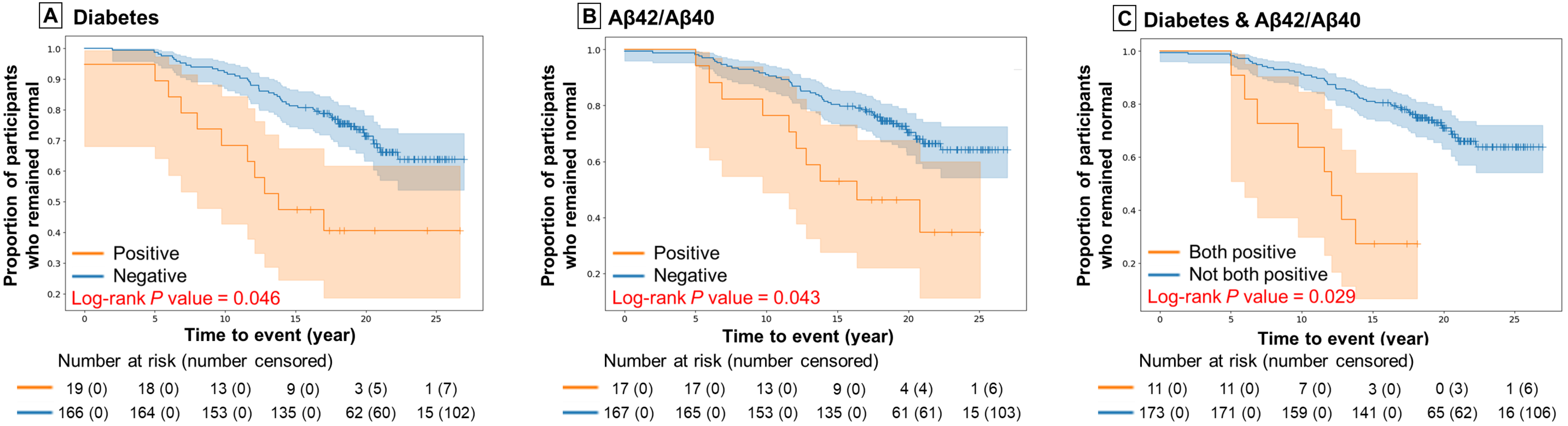

The Kaplan-Meier survival curves are separately displayed for the positive and negative groups for diabetes, CSF Aβ42/Aβ40 ratios, and both. Shadows indicate 95% confidence intervals for each group.

©2023 Uchida Y et al. *JAMA Network Open*.

eTable. Number of MRI Scans in Each Year

Year	No. of MRI scans
**National Institutes of Health**	
1995	2
1996	8
1997	11
1998	27
1999	19
2000	48
2001	40
2002	67
2003	68
2004	97
2005	34
**Johns Hopkins University**	
2015	97
2016	58
2017	88
2018	63
2019	95
2020	7
2021	51
2022	47
2023	24

©2023 Uchida Y et al. *JAMA Network Open*.

eReferences.

1. Albert M, Soldan A, Gottesman R, McKhann G, Sacktor N, Farrington L, Grega M, Turner R, Lu Y, Li S, et al. Cognitive changes preceding clinical symptom onset of mild cognitive impairment and relationship to ApoE genotype. *Curr Alzheimer Res*. 2014;11:773-784. doi: 10.2174/156720501108140910121920
2. Albert M, Zhu Y, Moghekar A, Mori S, Miller MI, Soldan A, Pettigrew C, Selnes O, Li S, Wang MC. Predicting progression from normal cognition to mild cognitive impairment for individuals at 5 years. *Brain*. 2018;141:877-887. doi: 10.1093/brain/awx365
3. Albert MS, DeKosky ST, Dickson D, Dubois B, Feldman HH, Fox NC, Gamst A, Holtzman DM, Jagust WJ, Petersen RC, et al. The diagnosis of mild cognitive impairment due to Alzheimer's disease: recommendations from the National Institute on Aging-Alzheimer's Association workgroups on diagnostic guidelines for Alzheimer's disease. *Alzheimers Dement*. 2011;7:270-279. doi: 10.1016/j.jalz.2011.03.008
4. McKhann GM, Knopman DS, Chertkow H, Hyman BT, Jack CR, Jr., Kawas CH, Klunk WE, Koroshetz WJ, Manly JJ, Mayeux R, et al. The diagnosis of dementia due to Alzheimer's disease: recommendations from the National Institute on Aging-Alzheimer's Association workgroups on diagnostic guidelines for Alzheimer's disease. *Alzheimers Dement*. 2011;7:263-269. doi: 10.1016/j.jalz.2011.03.005
5. Nishimaki K, Onda K, Ikuta K, Uchida Y, Mori S, Iyatomi H, Oishi K, Initiative tAsDN, Biomarkers tAI, aging Lfso. OpenMAP-T1: A Rapid Deep Learning Approach to Parcellate 280 Anatomical Regions to Cover the Whole Brain. *medRxiv*. 2024:2024.2001.2018.24301494. doi: 10.1101/2024.01.18.24301494
6. Djamanakova A, Tang X, Li X, Faria AV, Ceritoglu C, Oishi K, Hillis AE, Albert M, Lyketsos C, Miller MI, et al. Tools for multiple granularity analysis of brain MRI data for individualized image analysis. *NeuroImage*. 2014;101:168-176. doi: 10.1016/j.neuroimage.2014.06.046
7. Wu D, Ma T, Ceritoglu C, Li Y, Chotiyanonta J, Hou Z, Hsu J, Xu X, Brown T, Miller MI, et al. Resource atlases for multi-atlas brain segmentations with multiple ontology levels based on T1-weighted MRI. *NeuroImage*. 2016;125:120-130. doi: 10.1016/j.neuroimage.2015.10.042
8. Willemse EAJ, Tijms BM, van Berckel BNM, Le Bastard N, van der Flier WM, Scheltens P, Teunissen CE. Comparing CSF amyloid-beta biomarker ratios for two automated immunoassays, Elecsys and Lumipulse, with amyloid PET status. *Alzheimers Dement (Amst)*. 2021;13:e12182. doi: 10.1002/dad2.12182
9. Andreasen N, Sjögren M, Blennow K. CSF markers for Alzheimer's disease: Total tau, phospho-tau and Aβ42. *The World Journal of Biological Psychiatry*. 2003;4:147-155. doi: 10.1080/15622970310029912
10. Dakterzada F, Lopez-Ortega R, Arias A, Riba-Llena I, Ruiz-Julian M, Huerto R, Tahan N, Pinol-Ripoll G. Assessment of the Concordance and Diagnostic Accuracy Between Elecsys and Lumipulse Fully Automated Platforms and Innotest. *Front Aging Neurosci*. 2021;13:604119. doi: 10.3389/fnagi.2021.604119

©2023 Uchida Y et al. *JAMA Network Open*.
