# Supplementary material for: Acceleration of Brain Atrophy and Progression From Normal Cognition to Mild Cognitive Impairment
Source: JAMA Netw Open. 2024 Oct 30;7(10):e2441505. doi: 10.1001/jamanetworkopen.2024.41505 (PMC11525609; doi:10.1001/jamanetworkopen.2024.41505)
Supplement: Supplement 2. — Data Sharing Statement [file jamanetwopen-e2441505-s002.pdf]

Data Sharing Statement

Data
Data available: Yes
Data types: Data dictionary
How to access data: Data generated or analyzed during the study can be requested from the principal investigator, given appropriate ethical and data protection approvals and data transfer agreements.
When available: With publication

Supporting Documents
Document types: Statistical/analytic code
How to access documents: koishi2@jhmi.edu
When available: With publication

Additional Information
Who can access the data: Researchers whose proposed use of the data has been approved.
Types of analyses: For any purpose.
Mechanisms of data availability: With investigator support.
